# Contemporary insights and prospects on ferroptosis in rheumatoid arthritis management

**DOI:** 10.3389/fimmu.2024.1455607

**Published:** 2024-09-24

**Authors:** Hongyu Zhao, Qiumei Dong, Hao Hua, Hao Wu, Limei Ao

**Affiliations:** College of Traditional Chinese Medicine, Inner Mongolia Medical University, Huhhot, Inner Mongolia, China

**Keywords:** rheumatoid arthritis, ferroptosis, therapeutic targets, lipid peroxidation, iron metabolism, antioxidant defense

## Abstract

Rheumatoid arthritis (RA) is a common autoimmune disease characterized primarily by persistent synovial inflammation and joint destruction. In recent years, ferroptosis, as a novel form of cell death, has garnered widespread attention due to its critical role in various diseases. This review explores the potential mechanisms of ferroptosis in RA and its relationship with the pathogenesis of RA, systematically analyzing the regulatory role of ferroptosis in synovial cells, chondrocytes, and immune cells. We emphasize the evaluation of ferroptosis-related pathways and their potential as therapeutic targets, including the development and application of inhibitors and activators. Although ferroptosis shows some promise in RA treatment, its dual role and safety issues in clinical application still require in-depth study. Future research should focus on elucidating the specific mechanisms of ferroptosis in RA pathology and developing more effective and safer therapeutic strategies to provide new treatment options for RA patients.

## 1 Introduction

Rheumatoid Arthritis (RA), a disease with prominent features within the autoimmune disease spectrum, primarily manifests as synovial inflammation, which subsequently leads to degenerative changes in cartilage and bone erosion (1). In the early stages, the clinical manifestations of RA typically include morning stiffness, generalized discomfort, joint swelling, and tenderness. If not treated properly, it may lead to a systemic inflammatory response, affecting multiple organs such as the heart, liver, intestines, and muscles, ultimately resulting in joint deformity or disability, severely impacting the patient’s daily life. According to the 2010 Global Burden of Disease Study, RA ranks 42nd among 291 diseases in terms of disability (2), RA ranks 42nd in terms of disability among 291 diseases and is linked to 14% to 48% of depression cases (3). It is evident that RA has become a major disease impacting global human health levels.

The exact cause of RA has not yet been fully clarified. Current treatment mainly involves disease-modifying antirheumatic drugs (DMARDs), which are classified into traditional DMARDs (such as methotrexate [MTX], hydroxychloroquine, and sulfasalazine), biologics (such as TNF-α inhibitors, and IL-6 inhibitors), and new targeted drugs (such as JAK inhibitors like Baricitinib/Tofacitinib). MTX is widely considered the first-line treatment for RA worldwide over the past twenty years (4). When RA becomes more refractory, some biologic DMARDs may be used. Additionally, these DMARDs may be combined according to different disease needs, but due to issues such as drug resistance, such treatment approaches may lead to suboptimal outcomes (5, 6). Although nonsteroidal anti-inflammatory drugs (NSAIDs) and glucocorticoids (GCs) are used to treat RA, the former does not affect disease progression, and the latter has significant side effects with prolonged use. Hence, developing new therapeutic targets and effective drugs with new mechanisms is particularly important to address the clinical.

The concept of ferroptosis was first introduced in 2012 (7), It is a unique mode of programmed cell death distinct from apoptosis. It is characterized primarily by iron overload and lipid peroxidation, which are markedly different from other forms of programmed cell death. In cells undergoing ferroptosis, abnormal changes in mitochondrial structure can be observed, such as mitochondrial shrinkage, increased mitochondrial membrane density, and mitochondrial cristae degradation. Additionally, cell membrane rupture occurs in ferroptosis, while the morphology of the cell nucleus remains unchanged (8). The biochemical features of ferroptosis can be summarized as a deficiency in cysteine (CYS), depletion of glutathione (GSH), and inactivation of glutathione peroxidase 4 (GPX4), although the underlying mechanisms are not yet fully understood (9). As research progresses, ferroptosis has been confirmed to play a significant role in many biological processes. Recent studies have increasingly shown that ferroptosis plays a critical regulatory role in autoimmune and inflammatory diseases (10). Ferroptosis activator RAS-selective lethal small molecule 3(RSL3), for instance, can induce ferroptosis in synovial cells and worsen synovitis, and the pathways involved in the ferroptosis process can regulate RSL3 (11). Therefore, ferroptosis may be a potential target for future RA treatments.

This article aims to discuss the common features and potential links between ferroptosis and the pathogenesis of RA, and to evaluate the therapeutic applications of ferroptosis modulators in preclinical and clinical studies. It seeks to objectively assess the potential of ferroptosis in RA diagnosis and treatment, providing new research perspectives and clinical application strategies for RA diagnosis, treatment, and prognosis evaluation.

## 2 Overview of ferroptosis

### 2.1 Discovery of ferroptosis

Although the term “ferroptosis” was officially proposed in 2012 (7), some phenomena and changes associated with ferroptosis had been observed by researchers long before. In 1989, a similar form of neuronal cell death caused by cysteinedepletion was first reported, and this process was then referred to as “oxidative toxicity” (12). This neuronal cell death is induced by the excitotoxic glutamate through the inhibition of solute carrier family 7 member 11 (SLC7A11), which is an important component of the cysteine-glutamate antiporter system (system xc-). Later research confirmed that system xc- plays a critical role in the ferroptosis process. Additionally, oxidative toxicity is accompanied by the accumulation of reactive oxygen species (ROS), which is similar to ferroptosis. Some researchers even consider oxidative toxicity and ferroptosis to be two names for the same process (13). Over the subsequent years, the discovery of various ferroptosis inducers brought this unique form of programmed cell death into the public eye. Erastin, discovered in 2003, is a representative example that regulates cell death in an iron-dependent manner. Since then, new ferroptosis inducers, such as RSL3, have been continuously identified (14), With further research into ferroptosis inducers, scholars have discovered some distinct morphological features in cells induced by these agents, such as mitochondrial shrinkage, increased mitochondrial membrane density, and reduced or absent mitochondrial cristae. To date, the academic community has indeed found that certain substances can induce programmed cell death in an unknown manner with distinct morphological features. However, the key points and mechanisms of this type of cell death remain unclear, and there is still a lack of basic understanding of its mechanisms.

**Figure 1:**
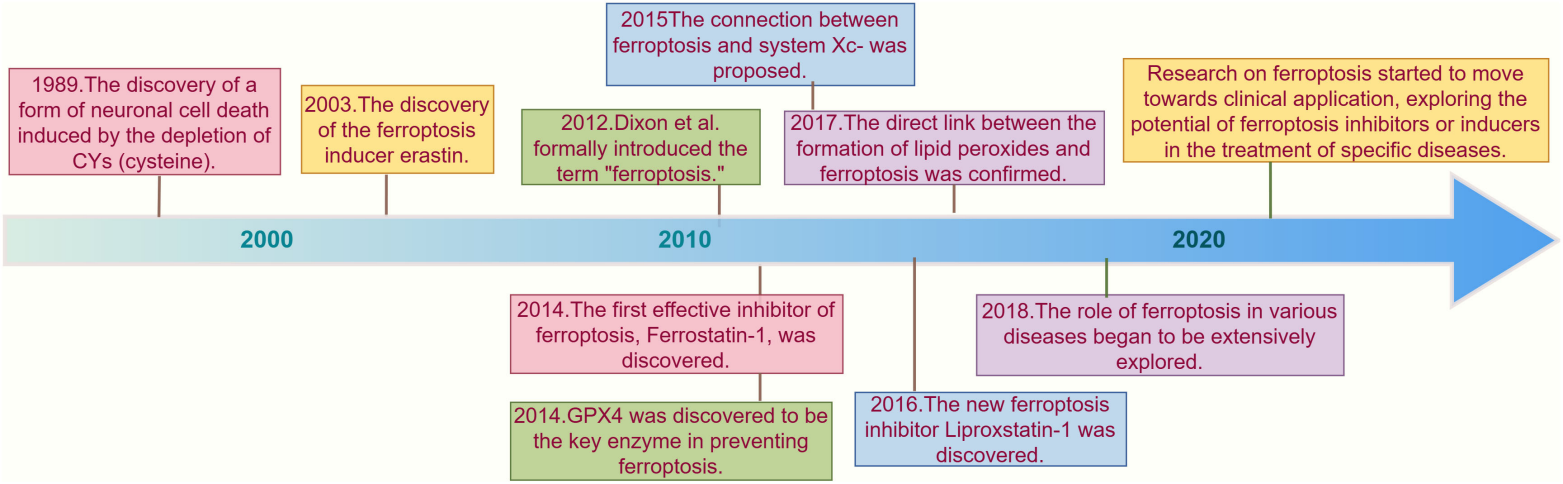
Timeline of key discoveries in ferroptosis research. This timeline illustrates the key discoveries and milestones in ferroptosis research from 1989 to the 2020s. In 1989, a phenomenon similar to ferroptosis was first reported, where neuronal cell death was induced by cysteine depletion (12) In 2003, the discovery of the ferroptosis inducer erastin marked a significant advancement in understanding this iron-dependent form of cell death (14). In 2012, the term "ferroptosis" was officially introduced by Stockwell and colleagues, who highlighted its role in cancer and neurodegenerative diseases (7). In 2014, GPX4 was identified as a critical regulator of ferroptosis, further deepening the understanding of its mechanisms (15). In 2015, research revealed that the tumor suppressor gene p53 can enhance ferroptosis sensitivity by downregulating SLC7A11 expression, suggesting potential therapeutic applications in cancer treatment (21). In 2016, Liproxstatin-1 was discovered as a novel ferroptosis inhibitor, providing researchers with new tools to study this cell death process (22). As the research progressed into the 2020s, the focus shifted towards the clinical applications of ferroptosis, particularly in cancer and neurodegenerative diseases (23–25). The research on ferroptosis has gradually entered a new era.

In 2012, Stockwell et al. (7) used erastin and RSL3 to treat RAS gene mutations and found that in the HT-1080 fibrosarcoma cell model carrying RAS mutations, erastin not only exerts antitumor effects by regulating mitochondrial voltage-dependent anion channels (VDAC) but also inhibits the function of SLC7A11, an important component of the system xc- system. This reduces the basic raw materials for intracellular GSH synthesis, leading to increased accumulation of iron-dependent lipid ROS and inducing cell death. They formally named this new type of cell death triggered by erastin as “ferroptosis” and pointed out that the activation of ferroptosis leads to non-apoptotic destruction of some cancer cells, while inhibiting this process can protect the organism from neurodegeneration. They also conducted preliminary exploration of the potential mechanisms and highlighted the relationship between ferroptosis and diseases. Thus, the concept of “ferroptosis” officially entered public view, although the specific deeper mechanisms of ferroptosis remain unclear. The appearance of this milestone paper led to a growing amount of research in this new field each year, with relevant targets being identified one by one. The Stockwell (15) discovered that GPX4 overexpression and knockout could regulate the lethality of 12 ferroptosis inducers, but not the lethality of 11 compounds with other mechanisms of lethality, confirming GPX4 as a key regulatory factor in ferroptosis. Research in 2015 (16) subsequently found that the tumor suppressor gene p53 can enhance cell sensitivity to ferroptosis by downregulating the expression of SLC7A11, thus inhibiting cysteine uptake. P53-3KR is a form of acetylation-deficient mutant p53 protein that retains its ability to regulate SLC7A11 expression and can induce ferroptosis in cells under ROS-induced stress, unrelated to cell cycle arrest, senescence, and apoptosis, revealing the potential application of ferroptosis in diseases. Shortly afterward, other research groups discovered that acyl-CoA synthetase long-chain family member 4 (NOX4) is a major mediator of ferroptosis (16, 17). In 2016, research found that activation of the p62-Keap1-NRF2 pathway can protect liver cancer cells from ferroptosis (18). In 2019, it was discovered that the FSP1-CoQ10-NAD(P)H pathway inhibits ferroptosis through a GPX4-independent mechanism (19). Since ROS accumulation is a significant feature of ferroptosis, and mitochondria are important sites of cellular metabolism and ROS production and targets, a team found that nitroxides targeting mitochondria could serve as effective inhibitors of ferroptosis (16). Mishima (20) identified vitamin K as an effective ferroptosis inhibitor through functional screening of vitamin compounds in GPX4-deficient mouse embryonic fibroblasts. With ongoing research, an increasing number of ferroptosis-related pathways have been discovered, including the transsulfuration pathway, the mevalonate (MVA) pathway, and the GTP cyclohydrolase-1 (GCH1) tetrahydrobiopterin (BH4) pathway. We have demonstrated the discovery and brief development timeline of ferroptosis as shown in **Figure 1**.

### 2.2 Three pathways of ferroptosis

#### 2.2.1 Lipid peroxidation system

Lipid peroxidation refers to the attack of free radical species, such as oxygen radicals, peroxide radicals, and hydroxyl radicals, on polyunsaturated fatty acids (PUFAs), leading to the accumulation of lipid peroxidation radicals and hydrogen peroxides (26). Increasing evidence shows that lipid peroxidation is a prerequisite for ferroptosis. Studies have found that the density of unsaturated fatty acids significantly affects the extent of lipid peroxidation, thereby determining the sensitivity to ferroptosis (27). PUFAs are converted into highly reactive lipid peroxides through a series of enzyme catalyses. Free PUFAs, including arachidonic acid (AA) and adrenal acid (ADA), are catalyzed into AA-CoA/AdA-CoA by acyl-CoA synthetase long-chain family member 4 (ACSL4). The activated lipid molecules are converted into AA-PE/AdA-PE by lysolipid phosphatidylcholine acyltransferase 3 (LPCAT3). Subsequently, lipid peroxidation occurs under the catalysis of lipoxygenase (LOXs) family proteins, producing excessive lipid peroxide (28). If these lipid peroxides are not removed in time, it will trigger a series of chain reactions, leading to the formation of a large number of products, disrupting membrane integrity, and ultimately causing the rupture of organelles and cell membranes, inducing ferroptosis (29). Since ACSL4 and LPCAT3 are involved in PUFAs catalysis, they have been identified as key regulatory targets for ferroptosis. Additionally, ALOX15 and ALOX12 from the LOX enzyme family are also targets of many ferroptosis inducers (30, 31).

#### 2.2.2 Iron metabolic pathways

Iron is one of the essential trace elements in the human body and is the most abundant trace element in the body. A deficiency of iron can lead to various iron-deficiency diseases, such as anemia and reduced immunity (32). However, despite its importance, iron must be maintained within a normal range. Excessive iron content, which results in increased total body iron levels, can lead to the accumulation of iron in tissues and organs, causing multi-organ dysfunction. Under normal circumstances, extracellular iron ions are mediated by transferrin (TF) and transferrin receptors (TFR). TF carrying Fe^3+^ binds to TFR on the target cell, transporting Fe^3+^ into the cell, where it is then reduced to Fe^2+^ by the metal reductase STEAP3, forming an unstable iron pool (LIP) inside the cell. Subsequently, intracellular Fe²^+^ is involved in various biological processes, including DNA synthesis, respiratory chain electron transport, oxygen transport and storage, and as a cofactor for various enzymes. However, the level of Fe²^+^ must be tightly regulated, as Fe²^+^ can directly catalyze the decomposition of hydrogen peroxide(H_2_O_2_) into hydroxyl radicals through the Fenton reaction, promoting ROS accumulation and lipid peroxidation (33). Therefore, if there is a disruption in iron metabolism, resulting in excess Fe^2+^ in cells, it can directly or indirectly induce cellular ferroptosis. Research has continuously shown that the expression of TF and TFR can directly influence the sensitivity of cells to ferroptosis (34, 35), indicating that iron overload due to disrupted iron metabolism is one of the key mechanisms triggering ferroptosis. Heme can be degraded by heme oxygenase-1 (HO-1), releasing Fe^2+^ which catalyzes lipid peroxidation reactions, thereby promoting the ferroptosis process. Studies have shown that the upregulation of HO-1 expression is closely related to the regulation of ferroptosis (36). Additionally, the autophagy of ferritin mediated by nuclear receptor coactivator 4 (NCOA4) contributes to maintaining iron homeostasis by converting ferritin to intracellular iron (37). This process can increase the release of free iron within the cell, and gene knockout or overexpression of NCOA4 can inhibit or induce ferroptosis (38). Therefore, various factors that cause disruption of iron metabolism can subtly affect ferroptosis.

#### 2.2.3 Antioxidant pathways

Excessive accumulation of PUFAs is a significant feature of ferroptosis. Further research has clarified that ferroptosis is characterized by the accumulation of lipid hydroperoxides induced by various oxidative stress stimuli, leading to cellular damage. The system Xc^-^-GSH-GPX4 pathway is currently the primary antioxidant defense mechanism against ferroptosis (39). System xc^-^ is a cystine-glutamate antiporter located on the cell membrane, consisting of a heterodimer formed by the light chain subunit SLC7A11 and the heavy chain subunit SLC3A2 (40), System xc^-^ facilitates the transport of glutamate out of the cell and cystine into the cell (41). Once inside the cell, cystine is oxidized to cysteine, which serves as a raw material for GSH synthesis. GSH is a small molecule active peptide, primarily composed of glutamate, cysteine, and glycine, and is a crucial antioxidant and free radical scavenger in the body. Numerous studies have shown that inhibition of system xc^-^ plays a key role in ferroptosis, protecting cells from damage caused by oxidative stress (42, 43). GPX4 is a selenium protein initially discovered through biochemical purification that can reduce lipid hydroperoxides (such as PLOOHs) to corresponding lipid alcohols, thereby mitigating lipid peroxidation-induced cellular damage. GSH, as a crucial cofactor for GPX4, plays a key role in its function (44, 45). When System xc- is inhibited, GSH synthesis decreases, leading to GPX4 inactivation and loss of antioxidant capability, which triggers cellular ferroptosis. As early as before ferroptosis was formally named, a study in 2003 found that conditional knockout of GPX4 in mouse primary fibroblasts resulted in extensive lipid peroxidation and non-apoptotic cell death (46). Subsequent research has found that both inhibition of GPX4 activity and direct knockout of the GPX4 gene lead to a sharp increase in lipid peroxidation levels, triggering the Fenton reaction, ROS accumulation, and worsening of cellular ferroptosis (47–49). The classic ferroptosis inducer erastin, as an effective system xc− inhibitor, can strongly and persistently inhibit system xc− through brief exposure to low concentrations of erastin, leading to GSH depletion and inducing ferroptosis (50). Therefore, targeting the Xc-GSH-GPX4 antioxidant pathway has gradually become a classic approach for regulating ferroptosis, with GPX4 being recognized as a core regulatory protein in ferroptosis.

### 2.3 Other pathways

**Figure 2:**
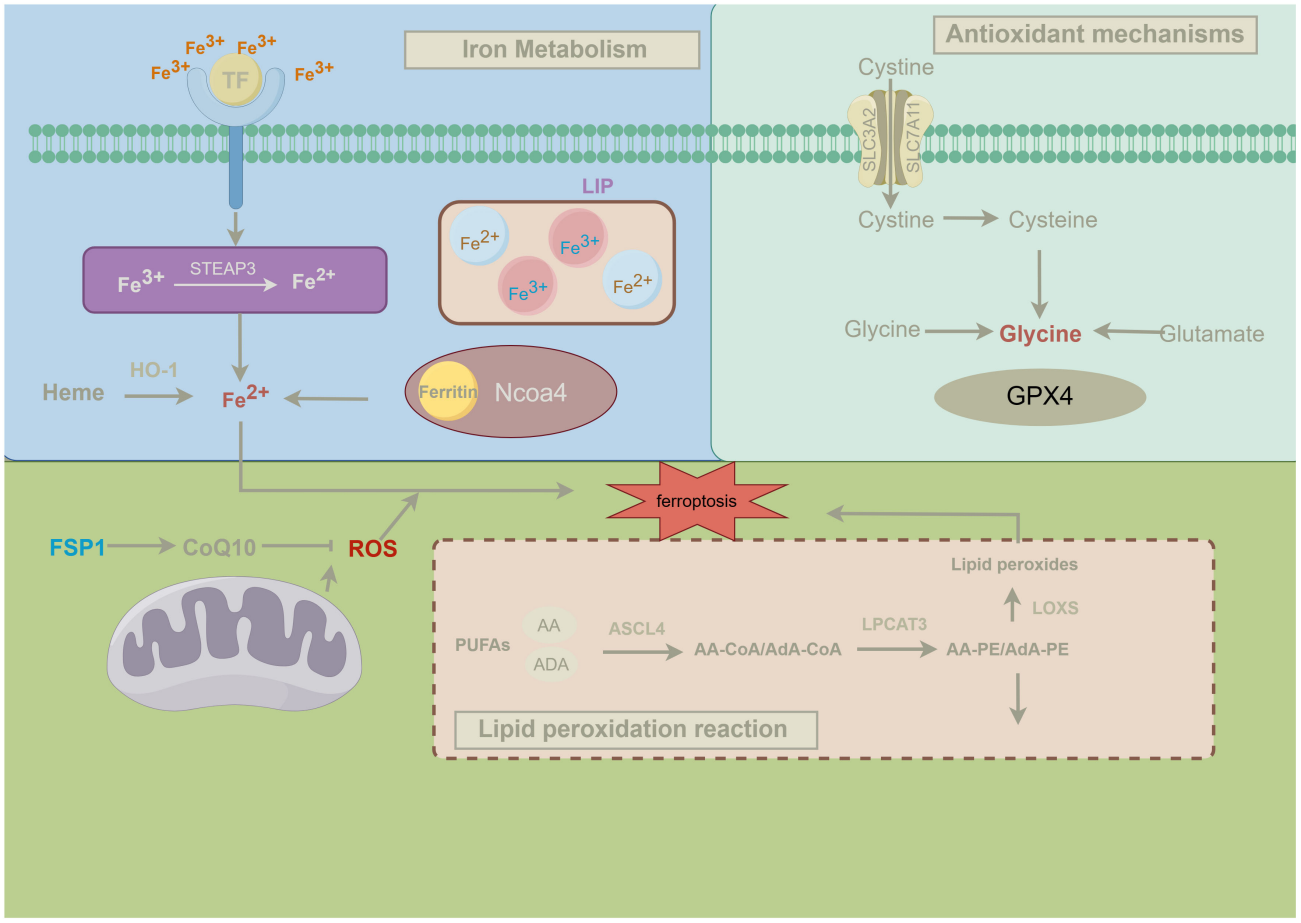
Mechanisms of ferroptosis. This figure illustrates the three classical pathways of ferroptosis and their regulatory mechanisms, details the key metabolic pathways involved in ferroptosis and their interactions. Firstly, iron metabolism is closely linked to ferroptosis. Transferrin (TF) transports Fe³⁺ into the cell, where it is reduced to Fe²⁺ by the enzyme STEAP3. Fe²⁺ can be stored in ferritin or released from heme via heme oxygenase-1 (HO-1), entering the labile iron pool (LIP) within the cell. During ferroptosis, NCOA4-mediated ferritinophagy releases Fe²⁺, which, through the Fenton reaction, promotes the generation of reactive oxygen species (ROS), triggering lipid peroxidation and ultimately leading to ferroptosis (53). Secondly, antioxidant mechanisms play a crucial role in regulating ferroptosis. The system xc⁻ antiporter imports extracellular cystine into the cell, which is reduced to cysteine and used in the synthesis of glutathione (GSH). Glutathione peroxidase 4 (GPX4) utilizes GSH to reduce lipid peroxides to non-toxic lipid alcohols, thereby preventing ferroptosis. When GPX4 function is compromised, lipid peroxides accumulate within the cell, leading to the onset of ferroptosis (54). Finally, lipid peroxidation is the central mechanism driving ferroptosis. Polyunsaturated fatty acids (PUFAs), such as arachidonic acid (AA) and adrenic acid (ADA), are esterified into phospholipids (e.g., AA-PE and AdA-PE) by ACSL4 and LPCAT3. These phospholipids are subsequently oxidized by lipoxygenases (LOXs) to form lipid peroxides, which, if not effectively neutralized by GPX4, initiate ferroptosis (7). Moreover, mitochondria play a significant role in ferroptosis, and the FSP1-CoQ10 axis serves as an alternative antioxidant mechanism independent of GPX4. This pathway reduces ROS levels through FSP1-mediated reduction of CoQ10, thereby preventing the propagation of lipid radicals and inhibiting lipid peroxidation and ferroptosis (52).

As research progresses, other antioxidant pathways have also been reported to independently suppress ferroptosis. Mitochondria play a crucial role in ferroptosis, especially in cysteine deprivation-induced ferroptosis, rather than GPX4 inhibition-induced ferroptosis. Cysteine deprivation leads to mitochondrial membrane potential hyperpolarization and accumulation of lipid peroxides. Inhibiting the mitochondrial tricarboxylic acid(TCA) cycle or electron transport chain (ETC) can mitigate mitochondrial membrane potential hyperpolarization, lipid peroxide accumulation, and ferroptosis (51). FSP1 (formerly known as AIFM2), a potent ferroptosis inhibitor, reduces coenzyme Q (CoQ) 10, a lipid-soluble radical-trapping antioxidant, to prevent the spread of lipid peroxidation and thereby inhibit ferroptosis. This indicates that FSP1 inhibits ferroptosis through a non-mitochondrial CoQ antioxidant system that operates parallel to GPX4 (52), revealing the complexity of the ferroptosis regulatory network and offering new directions for future ferroptosis research and therapeutic strategies. The following figure presents the brief mechanism of ferroptosis (**Figure 2**).

## 3 The role of ferroptosis in autoimmune diseases

Ferroptosis, a distinct form of programmed cell death dependent on iron accumulation and lipid peroxidation, has increasingly demonstrated its profound impact on various autoimmune diseases, including RA, as research continues to advance.

In systemic lupus erythematosus (SLE), the occurrence of ferroptosis is closely linked to abnormal immune responses and tissue damage. SLE is a complex, multisystem autoimmune disease characterized by the immune system’s erroneous attack on the body’s own healthy tissues, leading to widespread chronic inflammation and multi-organ damage. In recent years, increasing evidence suggests that ferroptosis may serve as a critical trigger in the pathogenesis of SLE, particularly in exacerbating inflammatory responses and promoting tissue damage. Studies have found that in SLE patients, ferroptosis not only acts as an alternative to apoptosis but also amplifies the immune response by releasing large amounts of DAMPs, such as high-mobility group box 1 (HMGB1), thereby aggravating the disease (10). HMGB1, as a key DAMP, can bind to specific receptors, thereby activating innate immune cells such as macrophages and dendritic cells. Under the stimulation of HMGB1, these immune cells rapidly initiate inflammatory responses, releasing large quantities of pro-inflammatory cytokines, including tumor necrosis factor-alpha (TNF-α), interleukin-6 (IL-6), and interleukin -1β(IL-1β). These cytokines play a crucial role in the pathogenesis of SLE, not only sustaining and exacerbating the inflammatory response but also contributing to further tissue damage. For example, TNF-α is one of the primary drivers of inflammation in SLE, exacerbating organ damage by inducing apoptosis and increasing vascular permeability. IL-6, on the other hand, promotes B cell differentiation and antibody production, which is closely associated with the generation and deposition of autoantibodies commonly observed in SLE patients (55).

Multiple sclerosis (MS) is a central nervous system (CNS) disorder characterized by the immune system’s erroneous attack on the myelin sheath, leading to impaired nerve conduction. Iron overload plays a critical role in MS, with research indicating that during ferroptosis, the excessive accumulation of iron and the formation of lipid peroxides in neurons result in severe neuroinflammation and axonal damage in MS patients (11). This process involves the excessive storage of iron through ferritin or the excessive entry of iron into cells via iron transport proteins, such as transferrin receptors and ferroportin 1. This leads to elevated levels of free iron within the cells. The free iron then generates a large amount of free radicals through the Fenton reaction, triggering lipid peroxidation, damaging cell membranes, and ultimately inducing ferroptosis (56). Ferroptosis inMS not only affects neurons but also has a profound impact on the function of immune cells, particularly the dysfunction of macrophages and microglia. Iron overload-induced ferroptosis leads to the release of large amounts of pro-inflammatory cytokines, such as TNF-α and IL-6, by these immune cells. These cytokines further exacerbate inflammation in the central nervous system, resulting in damage to the myelin sheath and neurons (57). The excessive activation of microglia exacerbates neural damage by releasing free radicals and pro-inflammatory cytokines, creating a vicious cycle that perpetuates inflammation and tissue destruction. This continuous cycle accelerates the progression of MS, further driving the disease’s advancement (11, 58).

Systemic sclerosis (SSc) is a rare autoimmune connective tissue disease characterized by widespread fibrosis of the skin and internal organs. Research indicates that ferroptosis may play a critical role in the pathogenesis of SSc, particularly in the pathological mechanisms associated with microvascular damage and oxidative stress. SSc patients often experience severe microvascular abnormalities, leading to local tissue hypoxia and reperfusion, which in turn generates large amounts of ROS. These ROS interact with intracellular iron, leading to the formation of lipid peroxides and ultimately inducing ferroptosis. Lipid peroxides not only directly damage cell membranes but also activate fibroblasts, accelerating the fibrosis of the skin and internal organs (59). Additionally, the ROS and lipid peroxides generated during ferroptosis can activate the immune system through various mechanisms, thereby exacerbating the inflammatory response. Specifically, the cellular damage caused by ferroptosis can release multiple pro-inflammatory mediators, which can recruit and activate macrophages and other immune cells. This activation enhances the local inflammatory response and further drives the progression of the disease (60). This suggests that regulating ferroptosis, along with its associated oxidative stress and inflammatory responses, could become a novel strategy for treating SSc.

In summary, ferroptosis plays a significant role in various autoimmune diseases, particularly in promoting inflammatory responses and tissue damage. Studies have shown that ferroptosis not only induces cell death through lipid peroxidation and oxidative stress but also amplifies immune responses by releasing DAMPs. These mechanisms are prominently observed in diseases such as systemic lupus erythematosus, multiple sclerosis, and systemic sclerosis. Similarly, ferroptosis may be crucial in regulating synovial cell proliferation and destruction, as well as processes related to iron metabolism and oxidative stress in the pathogenesis of RA. Therefore, further investigation into the role of ferroptosis in RA could reveal new therapeutic targets and strategies.

## 4 The role of ferroptosis in the pathogenesis of RA

The potential mechanisms of ferroptosis include the regulation of system xc-, GSH metabolism, GPX4 activity, and the excessive production of ROS. Recent studies have increasingly shown an association between the pathogenesis of RA and ferroptosis, indicating that ferroptosis is directly or indirectly involved in the onset and progression of RA. Therefore, elucidating its underlying molecular mechanisms may open new avenues for RA treatment.

### 4.1 Pathogenesis of RA

RA is a complex autoimmune disease involving multiple pathological changes in its onset and progression. The pathogenesis of RA is primarily driven by genetic susceptibility and environmental factors, leading to an immune system attack on the body’s own joint tissues, particularly resulting in inflammation of the synovial membrane.

Firstly, synovitis is one of the core pathological features of RA. Synovitis causes the proliferation of synovial tissue, leading to the formation of tumor-like synovial tissue, known as pannus. These abnormally proliferating synovial cells, such as fibroblast-like synoviocytes (FLS), not only increase significantly but also exhibit invasive behaviors similar to tumor cells, invading and destroying adjacent cartilage and bone tissue. Studies have shown that synovitis and pannus formation directly contribute to cartilage and bone destruction, and this invasive synovial tissue plays a critical role in the progression of RA (61).

Moreover, synovitis is not limited to the superficial synovial tissue but also affects the bone marrow. In many RA patients, bone marrow edema (BME) and inflammatory cysts can be detected through magnetic resonance imaging (MRI), and these inflammatory infiltrates are often precursors to bone destruction. The combined effects of synovitis, pannus formation, and bone marrow edema accelerate bone erosion and joint deformities (62).

As RA progresses, the overactivity of osteoclasts further exacerbates bone destruction. Under the influence of pannus, these cells become activated and begin resorbing bone tissue, leading to significant bone loss and joint deformities. Research indicates that bone destruction in RA is closely linked to the interaction between synovial tissue and osteoclasts, making this interaction a critical therapeutic target in RA (63).

In conclusion, the pathogenesis of RA involves multiple pathological processes, including synovitis, pannus formation, and bone destruction. These processes interact with each other, accelerating the progression of the disease and ultimately leading to the loss of joint function in patients.

### 4.2 Ferroptosis and RA

#### 4.2.1 Iron overload and oxidative stress

Iron metabolism dysregulation is a major cause of iron overload in RA patients. Under normal conditions, the absorption, storage, and excretion of iron are balanced. However, this balance is disrupted in RA patients. Hepcidin, a crucial hormone for regulating iron metabolism, is secreted by the liver. In inflammatory conditions, the expression of hepcidin is significantly increased by pro-inflammatory cytokines such as IL-6 and TNF-α. Moreover, ferritin, the primary storage form of intracellular iron, can be induced by inflammatory factors, leading to increased iron storage within cells. This increase in storage may cause iron overload, which in turn raises iron levels in synovial cells (64). In RA patients, the regulatory mechanisms for intestinal iron absorption may be altered, leading to increased iron intake. This change is typically associated with modifications in gut cell function during inflammatory states. Chronic inflammation may cause upregulation of iron transport proteins in intestinal epithelial cells, such as divalent metal transporter 1 (DMT1) and ferroportin (FPN). The elevated expression of these proteins can enhance intestinal iron absorption, allowing more iron to enter the bloodstream (65).

Iron overload significantly increases the production of ROS, particularly during the Fenton reaction. Excess iron reacts with H_2_O_2_ to generate hydroxyl radicals (·OH), which are highly reactive ROS. ·OH can attack intracellular macromolecules, including lipids, proteins, and DNA, leading to membrane damage, altered protein function, and genetic material destruction (65). The lipid peroxidation induced by OH leads to the oxidative degradation of membrane lipids, disrupting membrane integrity and function. Lipid peroxides not only damage cellular structures but also generate secondary ROS, exacerbating the oxidative stress environment. Under oxidative stress conditions, the antioxidant system in synovial cells is significantly inhibited. GSH, a major intracellular antioxidant, neutralizes ROS through GPX4. However, in the environment of iron accumulation and oxidative stress, GSH is rapidly depleted and cannot effectively remove excess ROS, leading to persistent oxidative stress within the cells. Additionally, the activity of antioxidant enzymes such as superoxide dismutase (SOD) and catalase (CAT) may be suppressed in RA patients. These enzymes are crucial for clearing excess ROS, and their reduced activity can intensify the oxidative stress environment within the cells (66).

#### 4.2.2 Proliferation of synovial cells and ferroptosis

One of the primary pathological features of RA is the abnormal proliferation of the synovium, with FLSbeing crucial in the disease process. These cells are involved in inflammation and also cause joint damage through invasive growth. In RA patients, the continuous activity of FLS increases the metabolic stress on synovial cells, enhancing their sensitivity to oxidative stress. This condition makes ferroptosis more likely in the iron-rich environment of synovial cells, potentially playing a key role in regulating synovial cell proliferation (53).

In RA patients, synovial cells often show disturbances in iron metabolism, with excessive iron accumulation leading to more active Fenton reactions, producing excessive ROS, and further promoting lipid peroxidation and ferroptosis (7). Concurrently, the decrease in intracellular GSH levels and the reduced activity of GPX4 are significant factors in inducing ferroptosis. Lower GPX4 activity causes increased lipid peroxidation in cell membranes, which triggers ferroptosis (15). In the RA synovial environment, the ongoing inflammatory state and oxidative stress intensify this process, positioning ferroptosis as a potential mechanism for controlling synovial cell proliferation.

In RA synovial cells, ferroptosis not only inhibits cell proliferation by directly inducing cell death but may also further affect RA progression by creating a negative feedback loop. Specifically, the damage-associated molecular patterns (DAMPs) produced during ferroptosis can trigger local immune responses, leading to the activation of macrophages and other immune cells in the synovium, which in turn release pro-inflammatory factors such as TNF-αand IL-6 (67). These inflammatory factors, while intensifying the inflammatory response, also exacerbate ferroptosis in synovial cells by increasing ROS production, thereby establishing a self-reinforcing inflammation-ferroptosis cycle. This cycle might, on one hand, curb excessive proliferation of synovial cells, but on the other hand, it could lead to further escalation of tissue damage and joint destruction (68). Furthermore, research has identified high expression of the ferroptosis-related gene TIMP1 in fibroblasts of RA synovial tissue, indicating its potential involvement in the proliferation and damage of RA synovial tissue (69).

#### 4.2.3 Lipid peroxidation and immune response

Lipid peroxidation is an oxidation reaction initiated by free radical or non-free radical mechanisms and is a key process in ferroptosis. It not only has a destructive effect on cell membrane integrity but also promotes the progression of RA by activating various inflammatory responses. Under oxidative stress, excessive production of ROS leads to lipid peroxidation reactions, generating lipid peroxides and other harmful products, which can cause cellular damage through various pathways (26). In RA, synovial cells and immune cells like macrophages are exposed to high levels of ROS in the inflammatory microenvironment, triggering significant lipid peroxidation reactions. These reactions not only directly damage these cells but also activate downstream inflammatory pathways through the production of metabolic byproducts, further exacerbating the inflammatory response in RA (70). Reactive metabolites produced during lipid peroxidation, such as Malondialdehyde (MDA) and 4-Hydroxy-2-nonenal(4-HNE), can be recognized as DAMPs by the immune system. These DAMPs bind to pattern recognition receptors (PRRs), activating immune cells like macrophages and dendritic cells, and inducing the production of pro-inflammatory cytokines such as TNF-α, IL-6, and IL-1β, thereby exacerbating the inflammatory response in RA (67).

In the pathological mechanisms of RA, the activation of the nuclear factor kappa-B(NF-κB) pathway is a central driver of inflammatory responses. Normally, NF-κB is bound to the inhibitor protein IκB, which maintains it in an inactive state. When exposed to external stimuli, IκB is phosphorylated by IκB kinase (IKK), leading to its degradation and the subsequent release of NF-κB. The activated NF-κB then enters the nucleus to initiate the transcription of pro-inflammatory genes, including TNF-α, IL-6, IL-1β, as well as various chemokines and adhesion molecules (71). Lipid peroxidation products, especially 4-HNE, can modify proteins by forming covalent bonds with cysteine, lysine, and histidine residues. IKK, a crucial regulatory protein in the NF-κB pathway, is particularly prone to modification by 4-HNE. Such modification can alter the conformation of IKK, keeping it in a persistently active state, which promotes the phosphorylation and degradation of IκB, and ultimately leads to the sustained activation of NF-κB (72). 4-HNE not only activates NF-κB by directly modifying proteins but also further boosts the generation of intracellular ROS. These ROS, through oxidative stress, further promote the activation of IKK, forming a positive feedback loop that perpetuates the activity of NF-κB. This continuous activation is a significant driver of chronic inflammation in RA (73).

#### 4.2.4 Ferroptosis in macrophages

Ferroptosis in immune cells not only affects the progression of inflammatory responses but also plays a crucial role in the onset and development of RA. Ferroptosis in immune cells, such as macrophages and neutrophils, can impact the inflammatory microenvironment of RA through multiple mechanisms.

In RA patients, macrophages are frequently in a highly activated state, and their proliferation and enhanced pro-inflammatory functions significantly contribute to synovial hyperplasia and joint erosion (74). In RA, macrophages are especially susceptible to ferroptosis due to their active metabolic state and increased levels of oxidative stress. Studies have found that ferroptosis in macrophages may exacerbate RA inflammation by releasing DAMPs that further activate other immune cells. For instance, HMGB1 can bind to receptors in the synovium, further driving the release of pro-inflammatory cytokines. These cytokines then promote additional ferroptosis, creating a detrimental feedback loop (8, 75). Ferroptosis in macrophages can lead to cell death, thereby reducing the macrophage count and lowering the release of pro-inflammatory factors. This reduction helps to alleviate the ongoing inflammatory response in the synovium. As the number of macrophages decreases, their pro-inflammatory stimulation of synovial cells also weakens. This reduction not only decreases the local concentration of inflammatory factors but also mitigates the abnormal proliferation and invasive behavior of synovial cells, which may help to prevent further joint damage to some extent.

#### 4.2.5 Ferroptosis of neutrophils and NETs

One important function of neutrophils in RA is to capture pathogens by forming neutrophil extracellular traps (NETs) (76). Recent studies have increasingly shown that ferroptosis plays a crucial role in this process. Ferroptosis promotes the formation of NETs by increasing ROS levels, thereby exacerbating the inflammatory response in RA. This mechanism not only complicates the role of NETs in RA but also reveals the multifaceted role of ferroptosis in the pathological process of RA.

**Figure 3:**
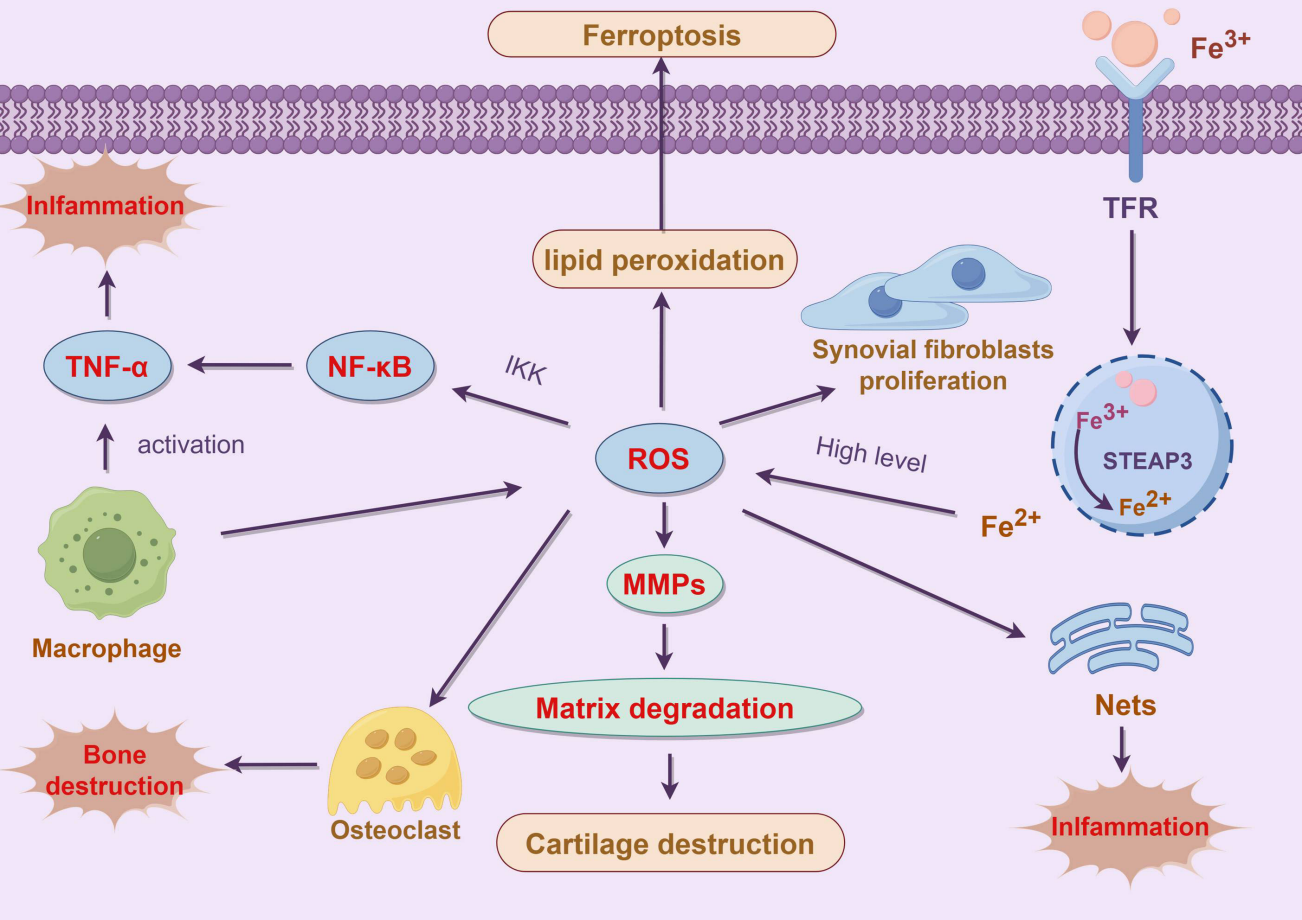
Role of ferroptosis in the pathogenesis of RA. This figure illustrates the pivotal role of ferroptosis in the pathogenesis of RA and its impact on inflammation and tissue damage. In RA patients, dysregulated iron metabolism leads to iron overload, which enhances the production of ROS via the Fenton reaction. This increase in ROS further promotes lipid peroxidation and cellular membrane damage, ultimately triggering ferroptosis (65). The abnormal proliferation of synovial cells in the RA microenvironment, accompanied by disturbed iron metabolism, exacerbates lipid peroxidation, thereby influencing ferroptosis and modulating the proliferation and inflammatory responses of synovial cells (67). Additionally, the accumulation of ROS during ferroptosis activates matrix metalloproteinases (MMPs), leading to extracellular matrix degradation and cartilage destruction (35). Ferroptosis-induced cellular damage and the formation of NETs further activate the NF-κB signaling pathway, increasing the expression of pro-inflammatory cytokines such as TNF-α, thereby amplifying the local inflammatory response (11). Ferroptosis plays a crucial role in the progression of RA through various mechanisms, including the regulation of synovial cell proliferation, enhancement of inflammatory responses, and promotion of bone and cartilage destruction. These processes collectively drive the pathological development of RA, making ferroptosis a significant focus in RA treatment and research.

NETs are mesh-like structures composed of DNA, histones, and antimicrobial proteins that are released by neutrophils when confronted with pathogens. These structures can capture and kill pathogens while restricting their spread. However, in chronic inflammatory diseases like RA, excessive NETs formation can be detrimental to tissue health. An overproduction of NETs not only fails to control infections effectively but also damages the host tissues. ROS is a crucial signal in promoting NETs formation. In RA, the accumulation of ROS during neutrophil ferroptosis drives these cells to release more NETs (76). Excessive NETs can further drive the inflammatory response and exacerbate the pathological progression of RA by activating other immune cells in the synovium (77, 78). Although NETs formation is a defensive mechanism of neutrophils against pathogens, excessive NETs in RA can act as an inflammatory amplifier. Firstly, components such as DNA and histones in NETs can serve as DAMPs. These DAMPs are recognized by macrophages and dendritic cells in the synovium, leading to their activation and the secretion of a large number of pro-inflammatory cytokines, which intensifies the local inflammatory response. Additionally, histones and antimicrobial proteins in NETs have cytotoxic effects, directly damaging synovial and cartilage cells, leading to tissue destruction. Furthermore, NETs formation promotes the infiltration of immune cells in the synovium, further advancing synovial hyperplasia and the expansion of the inflammatory response (79). The following figure illustrates the various mechanisms by which ferroptosis contributes to the progression of RA. These mechanisms include the regulation of synovial cell proliferation, enhancement of inflammatory responses, and the promotion of bone and cartilage destruction, thereby exacerbating the disease’s pathological development (**Figure 3**).

## 5 Crosstalk between ferroptosis and other forms of cell death and its significance in RA

In RA, ferroptosis exhibits complex interactions with other cell death modalities, such as apoptosis, necroptosis, and pyroptosis, underscoring its crucial role in the pathophysiology of the disease. As a novel form of regulated cell death driven by iron-dependent lipid peroxidation and oxidative stress, ferroptosis significantly affects synovial cell viability in RA. Moreover, its interplay with other cell death pathways can potentiate inflammatory responses and amplify tissue damage. Elucidating the specific mechanisms of ferroptosis in RA and its crosstalk with other forms of cell death could provide novel insights into the disease’s underlying pathology and offer promising targets for the development of innovative therapeutic interventions.

### 5.1 Ferroptosis and apoptosis

Ferroptosis and apoptosis are distinct forms of cell death, yet they can influence each other under certain pathological conditions. Apoptosis, a form of programmed cell death, is characterized by cell shrinkage, nuclear fragmentation, and apoptotic body formation. It is often regarded as a “clean” cell clearance mechanism that does not provoke an inflammatory response. In contrast, ferroptosis is an iron-dependent cell death modality driven by lipid peroxidation and oxidative stress, accompanied by the destruction of cell membranes.

In the pathological context of RA, synovial cells are exposed to various stressors, such as inflammatory cytokines (TNF-α, IL-6) and ROS, which can concurrently induce both apoptosis and ferroptosis. Specifically, oxidative stress can trigger ferroptosis by promoting lipid peroxidation, while also inducing apoptosis through mitochondrial membrane potential depolarization and cytochrome c release (53, 80). Thus, in RA, ferroptosis and apoptosis may exacerbate synovial tissue damage and inflammatory responses through shared oxidative stress pathways. Furthermore, the inhibition of GPX4 also increases cellular sensitivity to apoptotic signals. When GPX4 activity is reduced, levels of lipid peroxidation within the cell rise, promoting not only ferroptosis but also mitochondrial damage, thereby increasing the production of ROS and further triggering apoptosis (15). Therefore, modulating GPX4 activity could be a crucial strategy for simultaneously regulating both ferroptosis and apoptosis.

### 5.2 Ferroptosis and necroptosis

Necroptosis is a regulated form of necrotic cell death characterized by plasma membrane rupture and the release of intracellular contents, which subsequently triggers an inflammatory response. The occurrence of necroptosis is dependent on the activation of receptor-interacting protein kinase 1 (RIPK1), receptor-interacting protein kinase 3 (RIPK3), and mixed lineage kinase domain-like protein (MLKL). In the context of RA, inflammatory cytokines such as TNF-α can activate necroptosis through the RIPK1-RIPK3-MLKL signaling pathway, leading to cell death and exacerbation of inflammatory responses and tissue damage (81, 82).

There is a significant interaction between necroptosis and ferroptosis within the pathological context of RA. Research indicates that dysregulation of iron metabolism and resultant iron overload, frequently observed in RA patients, are strongly linked to the simultaneous activation of these two forms of cell death. Inflammatory cytokines can induce necroptosis through the RIPK1-RIPK3-MLKL signaling cascade while concurrently facilitating ferroptosis by elevating intracellular iron concentrations and augmenting oxidative stress via ROS production (83, 84). Under these conditions, necroptosis and ferroptosis are mutually reinforcing. During necroptosis, the activation of RIPK3 and translocation of MLKL leads to plasma membrane disruption and ROS production. These ROS not only exacerbate oxidative stress but also induce lipid peroxidation, increasing the likelihood of ferroptosis. Conversely, lipid peroxidation and the release of iron ions during ferroptosis further activate the RIPK1-RIPK3-MLKL pathway, accelerating the process of necroptosis (85).

### 5.3 Ferroptosis and pyroptosis

Pyroptosis is a highly inflammatory form of programmed cell death characterized by the activation of inflammasomes, such as NLRP3, leading to the cleavage of gasdermin D (GSDMD) and the formation of pores in the cell membrane, resulting in cell lysis (86). This process not only releases intracellular contents but also triggers a robust inflammatory response. In the pathological context of RA, synovial cells are subjected to persistent inflammatory stimuli, including TNF-α, IL-6, and other pro-inflammatory cytokines, which can activate the NLRP3 inflammasome and subsequently induce pyroptosis (87). Moreover, there exists a complex interplay between ferroptosis and pyroptosis, which further exacerbates inflammatory responses and tissue damage in RA.

In RA, DAMPs released during ferroptosis, such as HMGB1, can act as signaling molecules to further activate the NLRP3 inflammasome, thereby inducing pyroptosis. This interaction suggests that ferroptosis is not only a form of synovial cell death in RA but may also exacerbate local inflammatory responses by promoting pyroptosis (68). During pyroptosis, substantial amounts of inflammatory cytokines are released, leading to an increase in ROS levels (88). The elevated ROS enhances lipid peroxidation, thereby accelerating the process of ferroptosis. The interplay between cytokine release and ROS production establishes a positive feedback loop, amplifying inflammatory signaling and cell death. In the synovial tissues of RA, this positive feedback mechanism may not only exacerbate chronic inflammation but also further disrupt tissue architecture, thereby contributing to the progression of the disease.

The intricate crosstalk between ferroptosis and other forms of cell death, such as apoptosis, necroptosis, and pyroptosis, plays a crucial role in the pathophysiology of RA. A deeper understanding of these interactions may shed light on the underlying mechanisms of RA and potentially offer novel avenues for the development of targeted therapeutic strategies.

## 6 The application of ferroptosis in RA

### 6.1 Potential early treatment and diagnostic targets

Current research has demonstrated that early treatment for RA is crucial in preventing radiological progression of major joint damage. Ideally, starting treatment within 3-6 months after symptom onset can improve the success rate of disease remission and reduce joint damage and disability (89, 90). Early treatment requires early diagnosis; however, RA is challenging to diagnose in its early stages because its early signs and symptoms are similar to those of many other diseases. Diagnosis is difficult with just a single blood test or physical examination. Recent research has consistently confirmed the presence of iron accumulation and increased lipid peroxidation in RA-FLS. Consequently, modulating the oxidative microenvironment is increasingly recognized as a promising strategy for enhancing the effectiveness of RA treatment. However, research has discovered that hypoxia occurs in the pre-arthritis stage of RA, and even before clinically evident arthritis, hypoxia has already been present and is co-localized with early synovitis (91). Therefore, early intervention may protect normal synovial cells and maintain their physiological function, serving as an early prevention and treatment strategy for RA.

### 6.2 Application of drugs in RA

#### 6.2.1 Targeting the Xc-/GPX4 axis

Inhibiting the activity of the Xc- system can reduce the influx of cystine into cells, which further decreases intracellular glutathione synthesis and GPX4’s ability to scavenge peroxides, ultimately leading to cell death. Sulfasalazine, a DMARDs, can slow the progression of RA and control symptoms, thereby helping to protect joints from damage. *In vitro* studies have shown that sulfasalazine can significantly inhibit the xc- system and reduce the proliferation of lymphoma cells (92). RSL3 is a direct inhibitor of GPX4. It binds to the active site of GPX4, blocking its activity, and may also target the Xc-/GPX4 axis by affecting GSH levels associated with the xc- system, promoting ferroptosis in cells. This could have a positive impact on alleviating RA symptoms (93, 94). Imidazole erastin (IKE), a potent inhibitor of the xc- system, has been shown to reduce the number of fibroblasts in the synovium. Additionally, low-dose IKE combined with etanercept (a TNF antagonist) induces ferroptosis in fibroblasts and slows the progression of arthritis in the CIA model (95). The combination of TNF inhibitors and ferroptosis inducers may be a potential therapeutic strategy for RA. Galectin-1-derived peptide 3 (G1dP3) is a bioactive peptide derived from the galectin-1 domain with effective anti-inflammatory effects on RA-FLS. A recent study indicates that G1dP3 promotes ferroptosis in RA-FLS via the p53/SLC7A11 axis, suggesting its potential therapeutic role in RA (96). Additionally, the traditional Chinese medicine extract icariin has been found to activate the Xc-/GPX4 axis to inhibit ferroptosis, thereby protecting RA-FLS from LPS-induced cell death (97). The active component AB4 extracted from traditional Chinese medicine enhances cellular antioxidant capacity by upregulating GPX4 expression, further inhibiting the occurrence of ferroptosis (98), These studies provide a new direction for using traditional medicine to target ferroptosis in the treatment of RA.

#### 6.2.2 Regulation of iron metabolism

Iron metabolism pathways play a significant role in ferroptosis. Auranofin, an antirheumatic drug similar to sulfasalazine, has been found to have anticancer effects by directly altering the ferroptosis process in tumor cells (99). Research shows that high doses (25 mg/kg) of Auranofin induce iron toxicity by inhibiting the activity of thioredoxin reductase. At lower doses (5 mg/kg), Auranofin triggers hepcidin production, which reduces serum iron levels and transferrin saturation, thereby inhibiting ferroptosis in mice (100). TH1 is a crucial iron storage protein in ferroptosis. Research indicates that FTH1 is highly expressed in the synovium and RA-FLS. Treatment with glycine has been found to decrease FTH1 levels in RA-FLS (101), providing a new potential strategy for RA treatment.

#### 6.2.3 Inhibition of ROS

ROS can activate various cell types, including FLS and immune cells, thereby promoting the production of inflammatory cytokines and osteoclasts, and exacerbating joint damage. Relevant studies have explored the interaction mechanism between nuclear factor E2-related factor 2 (NRF2) and antioxidant response elements (ARE), and investigated how this mechanism promotes the production of cytotoxic electrophiles and ROS. The research indicates that effective binding of NRF2 to ARE significantly enhances these cellular stress responses (102, 103). MTX, a traditional medication for RA, has been shown to inhibit IL-6-induced ROS production, which might enhance its effectiveness in treating RA (104). Given that RA-FLS exhibit elevated levels of ROS and mitochondrial superoxide, a team has developed ROS-responsive nanomedicines to improve RA treatment in inflammatory environments. By assembling and preparing ROS-responsive berberine polymer micelles, the uptake of berberine in RA-FLS was significantly increased, and both *in vivo* and *in vitro* efficacy was enhanced (105). Concurrently, a team has designed ROS-responsive nanoparticles to specifically deliver dexamethasone (Dex) to inflamed joints and decrease the frequency of Dex administration. Both *in vivo* and *in vitro* studies show that these nanoparticles can be effectively internalized by activated macrophages, and Dex released from the nanoparticles significantly downregulates the expression of iRhom2, TNF-α, and BAFF, reducing joint swelling and cartilage destruction, thereby alleviating RA symptoms (106). The NK-1R antagonist aprepitant reduced TNF-α-induced NOX-4 expression and ROS production in RA-FLS (107, 108), indicating a novel potential role for NK-1R in RA-FLS.

#### 6.2.4 Potential therapeutic targets

In the serum of RA patients, the expression level of Silent Information Regulator 1 (SIRT1) is reduced, while the expression level of the transcription factor Yin Yang 1 (YY1) is elevated. Research has shown that in a lipopolysaccharide-induced synovial cell model, increased SIRT1 enhances cell viability and reduces levels of ROS and iron. At the molecular level, YY1 decreases SIRT1 protein expression by inhibiting the transcriptional activity of the SIRT1 gene. Furthermore, excessive expression of YY1 can partially reverse the effect of SIRT1 in downregulating iron levels in synovial cells (109). Elucidating the interaction between SIRT1 and YY1 and their effects on ferroptosis in RA synovial cells provides a scientific basis for new diagnostic and therapeutic targets for RA. Studies have investigated the ferroptosis-related mechanisms in RA, especially the effect of small extracellular vesicles (sEV) released by synovial cells on angiogenesis. The findings suggest that sEV released from synovial cells under ferroptosis conditions can promote angiogenesis. However, when the expression of miR-433-3p in these sEVs increases, they can inhibit angiogenesis induced by synovial cell sEVs (110). This research reveals the interaction between ferroptosis, miR-433-3p, and angiogenesis, offering a potential new target for RA therapy. **Table 1** summarizes recent targets, mechanisms, potential drugs, and their clinical research stages related to ferroptosis in RA, as well as the clinical significance of these targets in RA. These findings offer new perspectives on understanding the pathophysiological mechanisms of RA and provide potential molecular targets for the development of new treatment strategies.

**Table 1:** Relevant targets, mechanisms, potential drugs, and clinical research stages of ferroptosis in RA in recent years, as well as the clinical significance of these targets in RA.

Target Name	Mechanism of Action on Ferroptosis	Potential Drugs	Clinical Research Stage	Significance in RA	References
GPX4	Inhibition of Lipid Peroxidation to Prevent Ferroptosis	RSL3 Inhibitors, Vitamin E	Preclinical Research	Reduced GPX4 Activity Leads to Damage of Synovial Cells,Enhancing GPX4 Activity May Alleviate Joint Inflammation.	(101)
ACSL4	Increase Lipid Peroxidation to Accelerate Ferroptosis	Triacsin C	Preclinical Research	Inhibition of ACSL4 Can Reduce Inflammation and Cell Death.	(111)
Nrf2	Antioxidant Stress Reduction, Reducing Ferroptosis	Bardoxolone methyl	In Clinical Trials	Activating Nrf2 Can Reduce Oxidative Stress in RA and Protect Joint Tissues from Damage.	(112)
FSP1	Suppressing Ferroptosis, Maintaining Cell Survival	Ferroptosis-suppressor-protein 1	Preclinical Research	FSP1 Protects Joint Health by Reducing ROS Production and Suppressing Inflammatory Responses	(65)
CASP8	Pathways, Affecting Ferroptosis	Quercetin	Preclinical Research	CASP8 Overactivation May Lead to Apoptosis of Synovial Cells in RA; Quercetin Reduces Ferroptosis by Inhibiting CASP8	(113)
PTGS2 (COX-2)	Promote Inflammation and Oxidative Stress, Increasing Ferroptosis	NSAIDs, Specific Inhibitors	In Clinical Trials	High Expression of PTGS2 Is Associated with Worsening of RA; Inhibiting Its Activity Can Alleviate Symptoms and Protect Joints.	(114)
SLC7A11	Regulate Glutathione Metabolism to Prevent Ferroptosis	Erastin	Preclinical Research	Regulation of SLC7A11 Activity May Reduce Oxidative Damage in RA Synovial Cells and Alleviate Disease.	(115)
HIF-1α	Enhance Oxidative Stress to Promote Ferroptosis	Roxadustat	Preclinical Research	Overexpression of HIF-1α in RA is Associated with Inflammation and Joint Damage, Targeting Its Signaling Pathway May Alleviate RA Symptoms.	(116)

## 7 The double-edged role of ferroptosis in RA should not be ignored

Despite the potential of ferroptosis as a strategy for alleviating RA, existing research indicates that its potential effects in RA can be both beneficial and detrimental. Ferroptosis can induce the death of inflammatory cells, thereby reducing their infiltration in synovial tissue, which may help mitigate RA inflammation and tissue damage. On the other hand, ferroptosis may exacerbate joint damage by promoting the generation of free radicals and lipid peroxidation, which intensifies arthritis and pain. Thus, ferroptosis presents a dual-effect mechanism in therapeutic applications. Understanding the specific mechanisms of ferroptosis in RA and balancing its positive and negative effects is crucial for developing new treatment strategies. Furthermore, there is a significant gap in understanding and research regarding the potential toxicity of drugs and compounds that either inhibit or induce ferroptosis on other organs. Inducing ferroptosis to eliminate target cells might inevitably affect surrounding normal cells, potentially causing damage to healthy tissues. Conversely, inhibiting ferroptosis could impair the body’s ability to clear pathological cells, potentially increasing the risk of disease. Current studies on ferroptosis and RA are primarily focused on descriptive observations and outcome analysis, with less emphasis on the specificity of the ferroptosis process, accuracy of biomarkers, and *in vivo* quantification studies. Although several key characteristics and biomarkers associated with ferroptosis have been identified, specific detection and precise quantification of this process, particularly under physiological conditions, still face challenges. Future research needs to explore the specific role of ferroptosis in RA and how to effectively use ferroptosis regulation as a therapeutic strategy while minimizing potential toxicity risks. This is crucial for developing new treatment strategies and improving therapeutic outcomes.

## 8 Discussion

Ferroptosis, a unique mode of programmed cell death, has shown considerable potential in RA research. This process not only offers new therapeutic targets but also suggests innovative tools for disease diagnosis and monitoring. Despite current findings that elucidate the connection between ferroptosis and RA, the specific mechanisms within the RA pathological process demand further exploration.

In RA, ferroptosis potentially influences the disease’s onset and progression by regulating the survival of synovial cells, chondrocytes, and osteoclasts. Specifically, ferroptosis might mitigate RA’s inflammatory response by inhibiting synovial cell proliferation and reducing pro-inflammatory cytokine release. However, excessive lipid peroxidation and free radical production could exacerbate joint damage and pain. Thus, balancing the dual effects of ferroptosis in RA treatment is a critical focus for future research.

Although drugs that modulate ferroptosis, such as inhibitors or activators of the Xc-/GPX4 axis, have shown promise in preclinical studies, their clinical safety warrants careful evaluation. The impact of these drugs on non-target cells and their systemic side effects are priority areas for future investigation. Additionally, current deficiencies in the detection and quantification of ferroptosis-related biomarkers limit its widespread application in RA.

In summary, ferroptosis presents significant research value and potential clinical applications in RA. However, to ensure safe and effective therapeutic use, a deeper understanding of ferroptosis mechanisms in RA, optimization of related treatment strategies, and a comprehensive assessment of potential long-term risks are necessary. Future research should continue to explore the regulation of ferroptosis in RA, aiming to provide more refined therapeutic options for RA patients.
